# Short-Term Renal Effects of Add-On Levetiracetam in Client-Owned Dogs with Idiopathic Epilepsy Receiving Phenobarbital: A Four-Week Prospective Study

**DOI:** 10.3390/vetsci13090852

**Published:** 2026-08-23

**Authors:** Ubolbhorn Kankitpaisan, Chitasuda Ounprasert, Suporn Thongyuan, Nirut Suwanna

**Affiliations:** 1 Kasetsart University Veterinary Teaching Hospital, Bangkhen Campus, Faculty of Veterinary Medicine, Kasetsart University, Bangkok 10900, Thailand; ubolbhorn.ka@ku.th (U.K.); chitasuda.ou@ku.th (C.O.); 2 Department of Veterinary Public Health, Faculty of Veterinary Medicine, Kasetsart University, Nakhon Pathom 73140, Thailand; fvetspty@ku.ac.th; 3 Department of Companion Animal Clinical Sciences, Faculty of Veterinary Medicine, Kasetsart University, Bangkok 10900, Thailand

**Keywords:** canine idiopathic epilepsy, levetiracetam, phenobarbital, renal biomarkers, symmetric dimethylarginine (SDMA)

## Abstract

Levetiracetam (LEV) is commonly used as an adjunctive antiseizure medication (ASM) in dogs because of its favorable pharmacokinetic profile and limited hepatic metabolism. However, because LEV is predominantly eliminated through renal excretion, prospective clinical evidence regarding its effects on renal function in dogs remains limited. This prospective study evaluated short-term changes in renal biomarkers and urinalysis parameters in 12 client-owned dogs with idiopathic epilepsy (IE) receiving concurrent phenobarbital (PB) and adjunctive LEV. All 12 dogs were evaluated at baseline (day 0), day 14, and day 28. Renal assessment included serum symmetric dimethylarginine (SDMA), serum creatinine, blood urea nitrogen (BUN), phosphorus, albumin, and urinalysis; glomerular filtration rate (GFR) was not directly measured. Changes in SDMA over time were analyzed using the Friedman test, whereas serum creatinine, BUN, phosphorus, and albumin were analyzed using repeated-measures ANOVA with the Geisser–Greenhouse correction. No statistically significant changes were detected in SDMA, serum creatinine, BUN, phosphorus, or albumin during the 4-week study period. Urinalysis showed no consistent progressive abnormalities, including proteinuria, glucosuria, or urinary casts. Sedation was the most frequently observed clinical adverse event, occurring in 83% (10/12) of dogs, although it was generally mild and did not require treatment discontinuation. Overall, no detectable short-term deterioration in the assessed renal biomarkers or urinalysis parameters was identified during the 4-week study period in non-azotemic dogs receiving concurrent PB and adjunctive LEV. However, the absence of statistically significant or clinically detectable changes does not exclude subtle alterations in renal function or subclinical renal injury. Larger controlled studies with longer follow-up periods and direct assessment of GFR are warranted.

## Simple Summary

Levetiracetam (LEV) is a medication commonly added to other treatments to help control seizures in dogs with idiopathic epilepsy. Because LEV is removed from the body mainly through the kidneys, information about its effects on kidney function in dogs is important. In this study, 12 client-owned dogs receiving phenobarbital (PB) were given LEV as additional treatment and monitored for 4 weeks. Kidney-related blood and urine measurements showed no clinically important changes during the study period. However, these findings cannot rule out subtle kidney injury or changes that may not have been detected by the tests used in this study. Sedation was commonly observed, occurring in 10 of 12 dogs, but was generally mild and did not require treatment discontinuation. Because all dogs received both PB and LEV, the contribution of each medication to sedation could not be determined. Overall, the findings provide preliminary evidence that no clinically detectable deterioration in the kidney-related measurements assessed occurred during 4 weeks of combined PB and LEV treatment. Larger studies with longer follow-up periods are needed to further evaluate the effects of LEV on kidney function.

## 1. Introduction

Epilepsy is one of the most common chronic neurological disorders in dogs, with an estimated prevalence of approximately 0.6–0.75% in the general canine population [1,2,3].

Epileptogenesis is a complex process involving genetic and acquired factors that promote neuronal hyperexcitability and hypersynchronous activity through alterations in neurotransmission, ion channel function, neuroinflammation, and neuronal network organization [4]. Recurrent seizures can adversely affect quality of life and survival, and some affected dogs experience cluster seizures (CSs) or status epilepticus (SE), which represent clinically important and potentially life-threatening seizure emergencies [5,6]. Seizure pattern, together with age at seizure onset and interictal neurological examination findings, also provides important information for clinical assessment and diagnostic decision-making in dogs presenting with seizures [7].

Idiopathic epilepsy (IE) is a common form of epilepsy in dogs and encompasses epilepsy with a presumed genetic basis or epilepsy of unknown cause [8,9]. According to the International Veterinary Epilepsy Task Force (IVETF), IE is diagnosed based on clinical history, neurological examination, and exclusion of reactive and structural causes, with diagnostic confidence categorized according to established consensus criteria [8,9].

**Figure 1:**
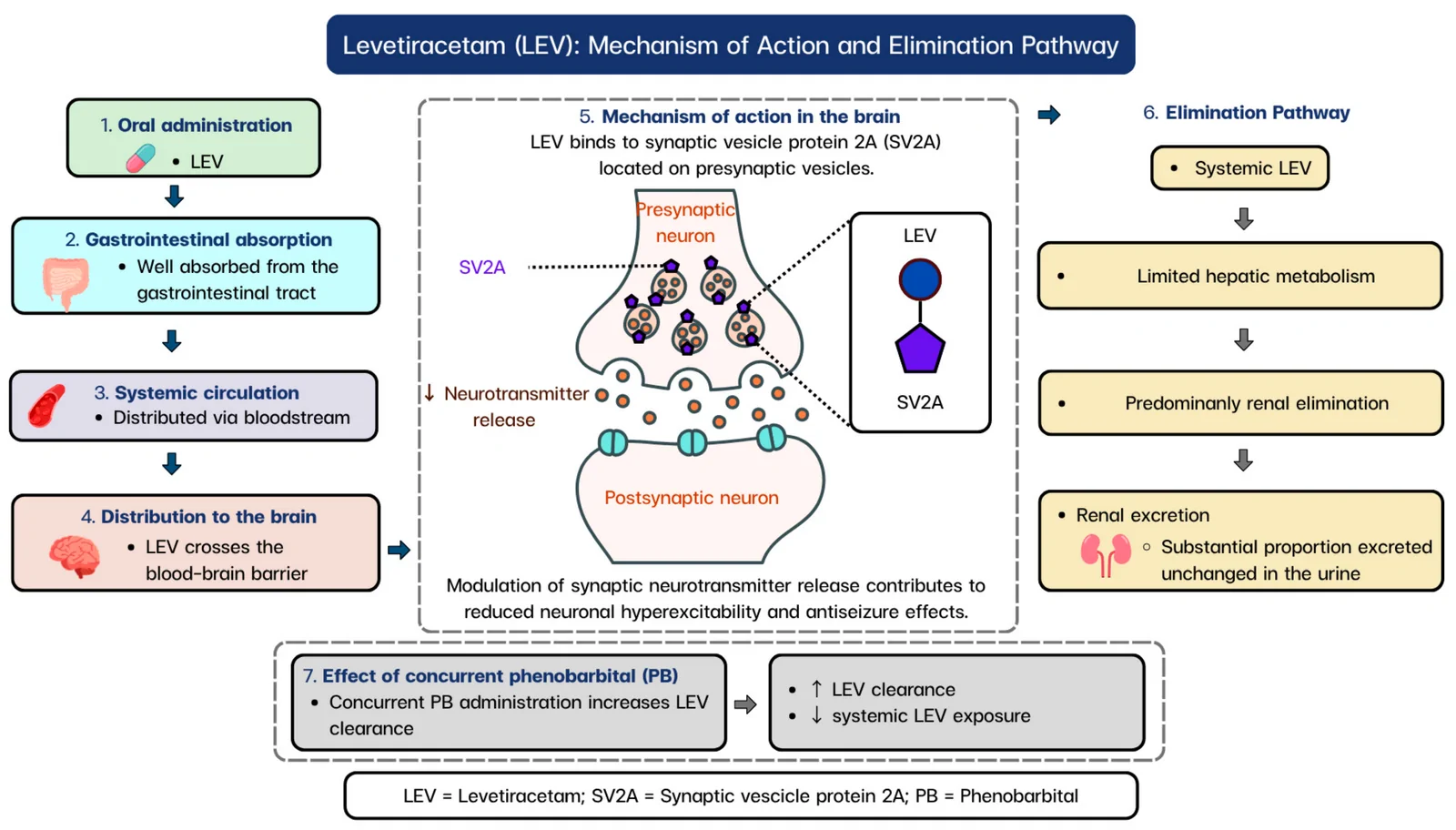
Schematic representation of the mechanism of action and elimination pathway of levetiracetam (LEV). Following oral administration and gastrointestinal absorption, LEV enters the systemic circulation, crosses the blood–brain barrier, and binds to synaptic vesicle protein 2A (SV2A) located on presynaptic vesicles. Binding to SV2A modulates synaptic neurotransmitter release, contributing to reduced neuronal hyperexcitability and antiseizure effects. LEV undergoes limited metabolism and is predominantly eliminated by renal excretion, with a substantial proportion excreted unchanged in the urine. Concurrent phenobarbital (PB) administration increases LEV clearance and reduces systemic LEV exposure. Arrows indicate the direction of LEV disposition and the relationships among the depicted pharmacokinetic and pharmacodynamic processes.

Phenobarbital (PB), potassium bromide (KBr), and, in some regions, imepitoin are commonly used antiseizure medications (ASMs) for canine IE. However, some dogs continue to experience recurrent seizures despite ASM treatment or develop treatment-related adverse effects, necessitating adjunctive therapy [6,10,11]. Levetiracetam (LEV), which binds to synaptic vesicle protein 2A (SV2A), is frequently used as an adjunctive ASM in dogs because of its favorable pharmacokinetic profile and tolerability [10,11,12,13,14,15,16]. LEV exhibits minimal protein binding and limited hepatic metabolism and is predominantly eliminated through renal excretion, with a substantial proportion excreted unchanged in the urine [13,14,15,16]. In healthy dogs, LEV has a relatively short elimination half-life of approximately 3–6 h, generally requiring administration every 8 h [6,13]. Concurrent PB administration may further alter LEV pharmacokinetics by increasing clearance and reducing systemic drug exposure [12,17]. The principal mechanism of action and elimination pathways of LEV are summarized schematically in Figure 1.

The predominant renal elimination of LEV provides a rationale for evaluating kidney function during treatment, particularly in dogs receiving long-term antiseizure therapy or those with underlying renal dysfunction. Kidney function in clinical veterinary practice is commonly assessed using serum creatinine, blood urea nitrogen (BUN), urine specific gravity (USG), and urinalysis [18,19]. Serum symmetric dimethylarginine (SDMA) provides an additional marker of glomerular filtration and has been incorporated into the International Renal Interest Society (IRIS) guidelines for the assessment of kidney disease in dogs [19,20,21,22]. Although SDMA may identify reductions in glomerular filtration rate (GFR) earlier than serum creatinine in some patients, renal biomarkers have inherent limitations and should be interpreted together with urinalysis and other clinical findings rather than in isolation [18,19,20,21,22].

Although LEV is generally considered well tolerated, information regarding its potential renal effects in dogs remains limited. Rare cases of acute kidney injury, including acute interstitial nephritis, have been reported in humans receiving LEV [23,24,25,26]. In veterinary medicine, previous studies have primarily focused on the pharmacokinetics, tolerability, and antiseizure effects of LEV, whereas prospective clinical data evaluating serial renal biomarkers and urinalysis during adjunctive LEV therapy remain scarce [10,11,12,13,14,15,16,17]. To the authors’ knowledge, prospective studies evaluating serial renal biomarkers together with urinalysis in client-owned dogs with IE receiving concurrent PB and adjunctive LEV are limited.

Therefore, the objective of the present prospective study was to evaluate short-term changes in renal biomarkers, serum biochemical parameters, and urinalysis findings in non-azotemic client-owned dogs with IE receiving adjunctive LEV together with PB over a 4-week period. We hypothesized that no clinically significant changes in the assessed renal parameters would occur during the study period. Given the limited sample size, short follow-up period, and concurrent PB administration, the study was designed to provide preliminary prospective data on short-term renal monitoring rather than to establish definitive renal safety or treatment efficacy.

## 2. Materials and Methods

### 2.1. Ethics

Ethical approval was granted by the Institutional Animal Care and Use Committee of Kasetsart University, Bangkok, Thailand (ACKU68-VET-107). Owners were informed in both written and verbal formats about the study objectives, data handling, and data privacy prior to enrollment. Written informed consent from each owner was obtained before data collection.

### 2.2. Study Population

An a priori power analysis was performed using G*Power version 3.1.9.7 (Heinrich Heine University, Düsseldorf, Germany) for a repeated-measures within-subjects design. Because prior prospective data specifically evaluating short-term changes in renal biomarkers during adjunctive LEV therapy in dogs were unavailable, an effect size (Cohen’s f) of 0.5 was specified a priori as an assumption for sample-size estimation. With an alpha level of 0.05 and a statistical power of 0.95, the estimated minimum sample size was 12 dogs. This calculation was intended to provide adequate power to detect relatively large within-subject changes and may not provide sufficient power to detect small-to-moderate changes.

Twelve client-owned dogs with IE were prospectively enrolled. All dogs had been receiving PB and experienced cluster seizure activity despite serum PB concentrations within the therapeutic range, prompting the addition of LEV as adjunctive antiseizure therapy. Baseline data collected at enrollment and retrieved from medical records included signalment (breed, sex, neuter status, and age at diagnosis), clinical history, seizure characteristics, previous antiseizure medication use, and the dosage and duration of PB treatment. Seizure data included seizure type and the acute cluster seizure burden immediately before LEV initiation. IE was diagnosed according to the IVETF consensus criteria based on clinical history, neurological examination, and exclusion of reactive and structural causes of seizures [27]. Dogs were excluded if they were receiving medications known to affect renal function, urine concentrating ability, glomerular filtration rate (GFR), or renal biomarkers, including potassium bromide (KBr), diuretics, angiotensin-converting enzyme inhibitors, angiotensin receptor blockers, calcium channel blockers (e.g., amlodipine), corticosteroids, or other potentially nephrotoxic medications. All dogs were non-azotemic at baseline, defined as a serum creatinine concentration <1.4 mg/dL according to the IRIS guidelines [22], with BUN concentrations within the laboratory reference interval. Serum SDMA concentrations were also within the laboratory reference interval at baseline, and urinalysis was performed to further characterize renal status.

All cases were managed at the Kasetsart University Veterinary Teaching Hospital, Bangkok, Thailand, and included both previously diagnosed cases and cases referred for ongoing treatment and management. Patient enrollment and sample collection were conducted prospectively between December 2025 and February 2026.

### 2.3. Levetiracetam Administration

Before LEV initiation, all dogs had been receiving PB at a dosage of approximately 3–4 mg/kg every 12 h. Serum PB concentrations were measured before LEV initiation and were within the therapeutic range (25–35 µg/mL) in all dogs. LEV was subsequently added as adjunctive antiseizure therapy because all dogs continued to experience cluster seizure activity despite therapeutic serum PB concentrations.

All dogs received oral LEV (Keppra^®^; UCB Pharma, Brussels, Belgium) as adjunctive antiseizure therapy at an initial dosage of 20–25 mg/kg every 8 h, based on previously recommended initial dosing regimens and clinical studies of immediate-release LEV in dogs [28,29]. Clinical examinations, seizure occurrence, and clinical adverse events were evaluated and recorded at each follow-up visit (days 14 and 28). Owner compliance with LEV administration was assessed through owner interviews and pill counts to verify adherence to the prescribed treatment regimen.

### 2.4. Study Design

This prospective study was conducted over 4 weeks, with evaluations performed on day 0, day 14, and day 28. Prior to initiation of the LEV treatment on day 0, all dogs underwent clinical evaluation and baseline laboratory testing consisting of complete blood count, serum biochemistry, serum SDMA measurement, and urinalysis.

All laboratory analyses were performed at the same diagnostic laboratory to ensure analytical consistency throughout the study period. Hematological analyses were performed using a Sysmex XN-1000 V (Sysmex Corporation, Kobe, Japan) and serum biochemical analyses were conducted using an ILab Taurus at the Kasetsart University Veterinary Diagnostic Laboratory, Bangkok, Thailand. SDMA concentrations were measured using an IDEXX Catalyst One^®^ SDMA Test, which utilizes an immunoassay-based methodology validated for use in dogs. Approximately 3–5 mL of urine was collected from each dog by cystocentesis or free catch (spontaneous voiding) and submitted to the veterinary diagnostic laboratory for urinalysis. Urinalysis was performed by laboratory personnel and included measurement of urine specific gravity (USG) using a refractometer, dipstick analysis using a Cobas Roche Urisys 1100 automated urine analyzer (Roche Diagnostics, Mannheim, Germany), and microscopic examination of urine sediment according to standard laboratory procedures.

Follow-up evaluations were performed on day 14 and day 28, during which seizure activity, clinical status, and laboratory parameters (hematology, serum biochemistry, SDMA, and urinalysis) were reassessed to monitor longitudinal changes and to evaluate any potential renal effects associated with the LEV administration.

### 2.5. Statistical Analysis

Statistical analyses were performed using GraphPad Prism (version 11.01; GraphPad Software, San Diego, CA, USA). Descriptive statistics were used to summarize the data. Categorical variables were expressed as frequencies and proportions with 95% confidence intervals (CIs). The normality of continuous variables, including SDMA, serum creatinine, BUN, albumin, and phosphorus, was assessed using the Shapiro–Wilk test. Normally distributed continuous variables were presented as mean ± standard deviation (SD), whereas non-normally distributed variables were presented as median and interquartile range (IQR). Confidence intervals were additionally calculated for the principal continuous outcomes; 95% CIs of the mean were reported for normally distributed variables, whereas nonparametric CIs of the median were reported for non-normally distributed variables.

Comparisons among day 0, day 14, and day 28 were performed using repeated-measures analysis of variance (ANOVA) for normally distributed variables. Sphericity was not assumed, and the Geisser–Greenhouse correction was applied. When a statistically significant overall effect of time was identified, Bonferroni-adjusted pairwise comparisons were performed. Non-normally distributed variables were analyzed using the Friedman test, followed by Dunn’s multiple-comparisons test when a statistically significant overall difference was identified. A *p*-value < 0.05 was considered statistically significant.

## 3. Results

### 3.1. Study Animals

Twelve client-owned dogs with IE received oral LEV as adjunctive therapy during the 4-week study period. Of these, nine (75%) fulfilled the IVETF Tier I diagnostic confidence level, whereas three (25%) fulfilled the Tier II diagnostic confidence level. The most common breed was the Siberian Husky (*n* = 4, 33.3%), followed by the Chihuahua (*n* = 2, 16.7%) and mixed-breed dogs (*n* = 2, 16.7%). The mean age at LEV initiation was 5.7 ± 1.37 years, and the mean body weight was 22.0 ± 14.2 kg. Three dogs (25%) were spayed females, whereas nine (75%) were neutered males. Before LEV initiation, all dogs had received PB therapy for at least 6 months and had therapeutic serum PB concentrations (25–35 µg/mL). Despite ongoing PB treatment, all dogs continued to experience cluster seizure activity, prompting the initiation of adjunctive LEV therapy (Table 1).

Three dogs fulfilled the IVETF Tier II diagnostic confidence level based on advanced diagnostic evaluation. Representative MR images from one of these dogs are provided in Supplementary Figure S1.

### 3.2. Clinical Signs

During the 4-week study period, 11 of 12 dogs had no recorded seizures, whereas one dog experienced a single focal seizure (Table 1), which was resolved spontaneously without further complications.

During adjunctive LEV therapy, the clinical adverse events noted were minor and well tolerated, as shown in Table 2. Sedation was the most frequently observed clinical adverse event, occurring in 10/12 dogs (83.3%). Other observed clinical adverse events included ataxia and decreased appetite, each reported in 2/12 dogs (16.7%). No cases of vomiting or behavioral changes were observed during the study period, and no severe or treatment-limiting adverse events were identified.

### 3.3. Renal and Biochemical Outcomes

**Figure 2:**
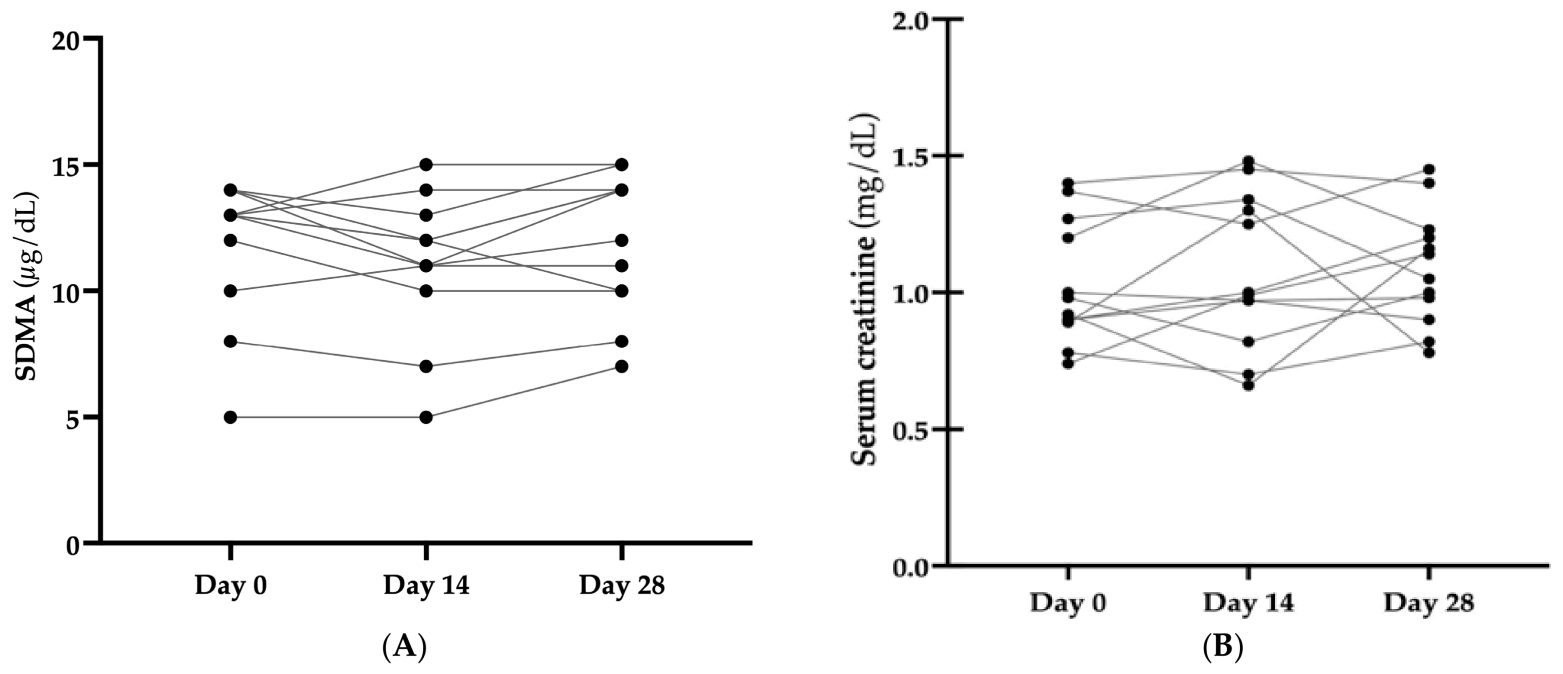
Individual changes in renal biomarkers over the 4-week treatment period. (**A**) Serum symmetric dimethylarginine (SDMA) concentrations and (**B**) serum creatinine concentrations at baseline (day 0), day 14, and day 28 after initiation of adjunctive levetiracetam treatment. Each point represents an individual dog, with lines connecting repeated measurements from the same dog.

No statistically significant changes were detected in renal biomarkers (SDMA and serum creatinine) or serum biochemical parameters (BUN, albumin, and phosphorus) throughout the 4-week study period (Table 3). SDMA did not satisfy the assumption of normality and was therefore analyzed using the Friedman test, which showed no statistically significant difference across the three assessment time points (χ^2^(2) = 5.415, *p* = 0.067). Repeated-measures ANOVA with the Geisser–Greenhouse correction similarly showed no statistically significant effect of time on serum creatinine (F(1.499, 16.49) = 0.553, *p* = 0.536), BUN, albumin, or phosphorus (Table 3). Individual changes in SDMA and serum creatinine concentrations across the three assessment time points are shown in Figure 2. Confidence intervals for the renal biomarkers and serum biochemical parameters at each assessment time point are presented in Supplementary Table S1. Additionally, three dogs underwent descriptive follow-up evaluation approximately 3 months after LEV initiation, with no clinically relevant changes observed in SDMA, serum creatinine, or other serum biochemical parameters.

No clinically significant changes were observed in urinalysis findings throughout the 4-week study period (Table 4). Urine specific gravity (USG) ranged from 1.010 to 1.042 during the study period. No dogs developed proteinuria, glucosuria, or urinary casts during the study period. Transitional epithelial cells were identified in two dogs at baseline but were not detected at subsequent evaluations.

## 4. Discussion

In the present study, no statistically significant changes were observed in renal biomarkers (SDMA and serum creatinine), serum biochemical parameters (BUN, phosphorus, and albumin), or urinalysis findings during the 4-week treatment period. These findings indicate that no clinically detectable deterioration in the assessed renal parameters occurred during short-term adjunctive oral LEV therapy in non-azotemic dogs with IE receiving concurrent PB. However, the absence of detectable abnormalities in SDMA, serum creatinine, and urinalysis does not exclude subclinical renal injury or subtle changes in renal function that may not have been detected by the methods used in this study. Although three dogs underwent extended follow-up evaluation approximately 3 months after LEV initiation without clinically significant biochemical changes, these observations should be interpreted cautiously because of the small number of cases and the descriptive nature of the extended follow-up.

SDMA was included because it is widely used as a renal biomarker in veterinary medicine and has been incorporated into the IRIS guidelines for the evaluation and staging of kidney disease in dogs [22]. Serum creatinine remains an important marker of kidney function but has limited sensitivity for detecting early reductions in GFR, whereas SDMA may detect reductions in GFR earlier in some patients [19,21]. Nevertheless, a recent systematic review demonstrated variability in the diagnostic performance of SDMA across studies, emphasizing that SDMA should not be interpreted as a standalone indicator of renal function [19,32]. In the present study, the lack of significant changes in SDMA, together with stable serum creatinine concentrations and the absence of clinically relevant urinalysis abnormalities, suggests that no detectable deterioration in renal function occurred during the 4-week observation period. However, these findings should not be interpreted as evidence that subclinical renal injury was definitively absent, particularly because direct measurement of GFR and specific urinary biomarkers of tubular injury were not performed [18,19].

Although USG varied among individual dogs, no consistent decrease was observed during the study period. Occasional low USG values, including values as low as 1.010, may have been influenced by concurrent PB therapy, as polyuria and polydipsia are recognized adverse effects of PB administration in dogs [6,29]. However, these low USG values were not accompanied by concurrent increases in SDMA or serum creatinine concentrations or by proteinuria, glucosuria, urinary casts, or other clinically relevant urinalysis abnormalities, and no consistent decrease in USG was observed over time. Transitional epithelial cells identified in two dogs at baseline were also not detected at subsequent evaluations. Taken together, these findings did not indicate a consistent pattern of progressive renal dysfunction based on the parameters assessed. Nevertheless, USG is influenced by multiple physiological and clinical factors, including concurrent medications, and should therefore be interpreted together with renal biomarkers, urinalysis findings, and serial patient assessments rather than in isolation [18,19].

Retrospective clinical data suggest that clinically meaningful changes in renal parameters may be more relevant in dogs with pre-existing kidney disease, whereas non-azotemic dogs generally exhibit minimal biochemical changes during LEV therapy [33]. This is consistent with the findings of the present study, which included only non-azotemic dogs. In dogs with pre-existing CKD, increases in renal biomarkers during LEV therapy should be interpreted cautiously, as changes in renal parameters may be influenced by the underlying kidney disease and cannot necessarily be attributed solely to LEV administration. Furthermore, because LEV is eliminated predominantly through renal excretion, impaired renal function may reduce drug clearance and increase systemic exposure, supporting consideration of dosage adjustment and closer clinical and laboratory monitoring in dogs with renal dysfunction [14,33,34]. Further prospective studies including dogs with varying degrees of kidney dysfunction are therefore needed to determine whether the renal effects and pharmacokinetics of LEV differ in this population.

Although treatment efficacy was not a primary objective of the present study, seizure outcomes were recorded as part of clinical monitoring. Eleven of the 12 dogs remained seizure-free during the 4-week treatment period, whereas one dog experienced a single focal seizure. These observations are broadly consistent with previous reports describing adjunctive LEV as a treatment option for dogs with pharmacoresistant epilepsy [10,11,35]. However, baseline seizure data represented acute cluster seizure burden immediately before LEV initiation, whereas seizure occurrence during treatment was recorded over the 4-week study period. Because these observation periods were not comparable and reliable monthly seizure-frequency data before enrollment were unavailable, the baseline and treatment seizure data are descriptive and should not be interpreted as a direct comparison of treatment efficacy. Furthermore, the short follow-up period, small sample size, absence of a control group, and study design preclude conclusions regarding the efficacy of LEV. The LEV dosing regimen used in the present study was consistent with previously recommended initial dosing regimens and those evaluated in clinical studies of immediate-release LEV in dogs [28,29]. Concurrent PB administration should be considered when interpreting both the dosing regimen and seizure outcomes, as PB has been shown to increase LEV clearance, shorten its elimination half-life, and reduce systemic drug exposure [12,17]. Therefore, higher LEV doses may be required in some dogs receiving concurrent PB. Nevertheless, the recommended initial dosing regimen was used in the present study rather than empirically increasing the dose solely based on concurrent PB administration, as the primary objective was to evaluate short-term renal parameters rather than to optimize LEV pharmacokinetics or antiseizure efficacy.

Treatment tolerability is an important consideration when evaluating adjunctive antiseizure therapy because both seizure burden and treatment-related adverse effects may influence patient welfare and owner quality of life [11,36]. Sedation was the most frequently observed clinical adverse event in the present study, occurring in 83.3% of dogs receiving concurrent PB and adjunctive LEV therapy. Sedation has also been reported in previous studies evaluating adjunctive LEV therapy in dogs [10,36]. Although sedation was generally mild and did not require treatment discontinuation, the absence of a PB-only control group prevents attribution of this finding specifically to LEV. The observed sedation may therefore reflect the effects of PB, LEV, or their combined administration.

Human clinical studies suggest that clinically significant renal adverse effects associated with LEV are uncommon despite its predominant renal elimination [23]. Rare cases of acute interstitial nephritis and acute kidney injury have been reported, including events occurring within days to weeks after treatment initiation [24,25,26]. Therefore, the 4-week follow-up period in the present study provided an opportunity to evaluate clinically detectable early changes in the renal parameters assessed, but it was not sufficient to characterize delayed or chronic renal effects. Moreover, the absence of abnormalities in conventional renal biomarkers and urinalysis cannot exclude mild or subclinical renal injury. Continued renal monitoring may therefore be appropriate during LEV therapy, particularly in patients with pre-existing renal dysfunction.

To the authors’ knowledge, few prospective veterinary studies have evaluated serial SDMA measurements together with urinalysis during adjunctive LEV therapy in client-owned dogs. In the present study, no clinically significant changes were detected in the assessed renal biomarkers or urinalysis findings over the 4-week observation period. These results provide preliminary prospective data regarding short-term renal monitoring during adjunctive LEV therapy in non-azotemic dogs receiving concurrent PB. However, they should be interpreted as an absence of detectable changes in the parameters assessed rather than definitive evidence of an absence of renal injury.

A strength of the present study is that the diagnosis of IE was established according to the IVETF consensus criteria. Of the 12 enrolled dogs, nine fulfilled the Tier I diagnostic confidence level and three fulfilled the Tier II diagnostic confidence level, providing a well-characterized study population with a high diagnostic confidence for idiopathic epilepsy [8,27].

The present study has several limitations. First, the sample size was relatively small, and the priori sample-size calculation was based on an assumed large effect size (Cohen’s f = 0.5) because prior prospective data suitable for estimating the expected effect size were unavailable. Consequently, the study may have been underpowered to detect small-to-moderate changes in renal biomarkers, increasing the risk of a Type II error. Therefore, the absence of statistically significant differences should not be interpreted as evidence of an absence of renal effects. The reported confidence intervals provide additional information regarding the precision of the estimates; however, the small sample size should be considered when interpreting these findings. Second, the follow-up period was limited to 4 weeks and therefore may not have captured potential long-term renal effects associated with LEV administration. Third, although three dogs underwent extended follow-up evaluation for approximately 3 months without clinically significant biochemical changes, the small number of cases and descriptive nature of these observations limit their interpretation. Fourth, all enrolled dogs were non-azotemic, which may limit the generalizability of these findings to dogs with pre-existing or more advanced kidney disease. Fifth, the study did not include a PB-only control group. Because all enrolled dogs were receiving concurrent PB therapy, the independent effects of LEV could not be fully distinguished from those of PB or from potential interactions between PB and LEV. Therefore, the observed renal findings should be interpreted in the context of combined PB and LEV therapy rather than being attributed solely to LEV. Finally, renal function was assessed using conventional renal biomarkers and urinalysis rather than direct measurement of GFR. Although direct measurement of GFR is considered the reference standard for assessing renal function, its use remains technically demanding and is not routinely feasible in clinical veterinary practice [19,22]. Accordingly, the present findings should be considered preliminary and require confirmation in larger prospective studies with longer follow-up periods, appropriate control groups, inclusion of dogs with varying degrees of renal dysfunction, and, where feasible, direct measurement of GFR.

## 5. Conclusions

Adjunctive LEV therapy was generally well tolerated in the client-owned dogs with IE receiving concurrent PB included in this study. No clinically significant changes were detected in the assessed renal biomarkers, serum biochemical parameters, or urinalysis findings during the 4-week treatment period. However, these findings do not exclude subclinical renal injury or subtle changes in renal function, and the absence of a PB-only control group prevents attribution of the observed findings specifically to LEV. Overall, these results provide preliminary evidence that no clinically detectable deterioration in the assessed renal parameters occurred during short-term adjunctive LEV therapy in non-azotemic dogs receiving concurrent PB. Larger prospective controlled studies with longer follow-up periods, inclusion of dogs with varying degrees of renal dysfunction, and more direct assessments of renal function are warranted.

## Figures and Tables

**Table 1:** Baseline characteristics, cluster seizure burden before LEV initiation, and seizures observed during the 4-week treatment period.

Case	Breed	Age at LEV Initiation (Years)	Sex	Cluster Seizure Burden Immediately Before LEV Initiation	LEV Dose (mg/kg q8h)	Seizures Observed During LEV Treatment (4 Weeks)
1	Siberian Husky	7	MN	5 seizures/16 h	22.7	0
2	Siberian Husky	5.8	MN	4 seizures/16 h	21.4	0
3	Siberian Husky	5	MN	3 seizures/8 h	23.4	0
4	Siberian Husky	7	FS	3 seizures/10 h	22.5	0
5	Chihuahua	5.4	FS	4 seizures/18 h	20.8	1 seizure
6	Chihuahua	6	FS	3 seizures/4 h	20.8	0
7	Mixed breed	7.8	MN	5 seizures/24 h	25.0	0
8	Mixed breed	5	MN	4 seizures/12 h	24.3	0
9	Golden Retriever	7	MN	3 seizures/12 h	24.3	0
10	Beagle	3.1	MN	3 seizures/4 h	21.7	0
11	French Bulldog	4	MN	5 seizures/20 h	23.4	0
12	Yorkshire Terrier	5	MN	4 seizures/4 h	22.2	0

FS = female spayed; MN = male neutered. Baseline cluster seizure burden represents the number of seizures occurring within the specified time interval immediately before LEV initiation. All dogs experienced cluster seizure activity that prompted the addition of LEV to their existing antiseizure treatment. Seizures during treatment represent those recorded over the 4-week study period. Because baseline cluster seizure burden and seizures during treatment were recorded over different observation periods, these data are presented descriptively and should not be interpreted as a direct comparison of treatment efficacy.

**Table 2:** Clinical adverse events observed in 12 dogs during adjunctive levetiracetam therapy.

Adverse Effects in Dogs	Frequency (%)	95% CI (%)
Sedation	10 (83.3%)	51.6–97.9
Ataxia	2 (16.7%)	2.1–48.4
Decreased appetite or anorexia	2 (16.7%)	2.1–48.4
Vomiting	0 (0%)	0.0–26.5
Behavioral changes	0 (0%)	0.0–26.5

**Table 3:** Renal biomarkers and serum biochemical parameters during the 4-week adjunctive levetiracetam treatment period.

Variable	Reference Interval (Unit)	Day 0	Day 14	Day 28	*p*-Value
SDMA	0–14 µg/dL	13.00 (10.50–13.75)	11.50 (10.25–12.75)	13.00 (10.00–14.00)	0.067
BUN	8–28 mg/dL	17.92 (4.68)	17.50 (6.86)	20.25 (6.69)	0.188
Serum creatinine	0.5–1.7 mg/dL	1.03 (0.22)	1.08 (0.28)	1.09 (0.21)	0.536
Albumin	2.3–3.1 g/dL	3.43 (0.47)	3.29 (0.28)	3.32 (0.39)	0.207
Phosphorus	2.9–5.3 mg/dL	3.79 (0.51)	3.77 (0.35)	3.85 (0.45)	0.835

Data are presented as mean (SD) for normally distributed variables and median (IQR) for non-normally distributed variables. SDMA was analyzed using the Friedman test, whereas BUN, serum creatinine, albumin, and phosphorus were analyzed using repeated-measures ANOVA with the Geisser–Greenhouse correction. *p*-values represent overall comparisons across day 0, day 14, and day 28. Reference intervals for SDMA were based on the IDEXX Catalyst SDMA Test reference interval [30]. Reference intervals for BUN, serum creatinine, albumin, and phosphorus were based on those used by the Kasetsart University Veterinary Diagnostic Laboratory, adapted from Duncan & Prasse’s Veterinary Laboratory Medicine: Clinical Pathology [31]. SDMA = symmetric dimethylarginine; BUN = blood urea nitrogen; SD = standard deviation; IQR = interquartile range.

**Table 4:** Urinalysis findings during the 4-week adjunctive levetiracetam treatment period.

Parameter	Day 0	Day 14	Day 28
USG (range)	1.010–1.042	1.015–1.042	1.010–1.035
Proteinuria	0 (0%)	0 (0%)	0 (0%)
Glucosuria	0 (0%)	0 (0%)	0 (0%)
Epithelial cells	2 (16.7%)	0 (0%)	0 (0%)
Casts	0 (0%)	0 (0%)	0 (0%)

Data are presented as the range for urine specific gravity (USG) or as the number (%) of dogs with positive findings.

## Data Availability

The original contributions presented in this study are included in the article/Supplementary Material. Further inquiries can be directed to the corresponding author.

## Supplementary Materials

The following supporting information can be downloaded at: https://www.mdpi.com/article/10.3390/vetsci13090852/s1, Figure S1: Representative magnetic resonance (MR) images from one dog fulfilling the IVETF Tier II diagnostic confidence level; Table S1: Confidence intervals for renal biomarkers and serum biochemical parameters during the 4-week treatment period.

## Abbreviations

The following abbreviations are used in this manuscript:

ASMs	Antiseizure medications
AKI	Acute kidney injury
BUN	Blood urea nitrogen
CKD	Chronic kidney disease
GFR	Glomerular filtration rate
IE	Idiopathic epilepsy
IRIS	International Renal Interest Society
IVETF	International Veterinary Epilepsy Task Force
KBr	Potassium bromide
LEV	Levetiracetam
PB	Phenobarbital
SDMA	Symmetric dimethylarginine

## References

[1] Volk H.A. (2015). International Veterinary Epilepsy Task Force Consensus Reports on Epilepsy Definition, Classification and Terminology, Affected Dog Breeds, Diagnosis, Treatment, Outcome Measures of Therapeutic Trials, Neuroimaging and Neuropathology in Companion Animals. BMC Vet. Res..

[2] Kearsley-Fleet L., O’Neill D.G., Volk H.A., Church D.B., Brodbelt D.C. (2013). Prevalence and Risk Factors for Canine Epilepsy of Unknown Origin in the UK. Vet. Rec..

[3] Heske L., Nødtvedt A., Jäderlund K.H., Berendt M., Egenvall A. (2014). A Cohort Study of Epilepsy among 665,000 Insured Dogs: Incidence, Mortality and Survival after Diagnosis. Vet. J..

[4] Hui Yin Y., Ahmad N., Makmor-Bakry M. (2013). Pathogenesis of Epilepsy: Challenges in Animal Models. Iran. J. Basic Med. Sci..

[5] Charalambous M., Muñana K., Patterson E.E., Platt S.R., Volk H.A. (2024). ACVIM Consensus Statement on the Management of Status Epilepticus and Cluster Seizures in Dogs and Cats. J. Vet. Intern. Med..

[6] Bhatti S.F., De Risio L., Muñana K., Penderis J., Stein V.M., Tipold A., Berendt M., Farquhar R.G., Fischer A., Long S. (2015). International Veterinary Epilepsy Task Force consensus proposal: Medical treatment of canine epilepsy in Europe. BMC Vet. Res..

[7] Armaşu M., Packer R.M., Cook S., Solcan G., Volk H.A. (2014). An exploratory study using a statistical approach as a platform for clinical reasoning in canine epilepsy. Vet. J..

[8] Berendt M., Farquhar R.G., Mandigers P.J., Pakozdy A., Bhatti S.F., De Risio L., Fischer A., Long S., Matiasek K., Muñana K. (2015). International Veterinary Epilepsy Task Force Consensus Report on Epilepsy Definition, Classification and Terminology in Companion Animals. BMC Vet. Res..

[9] Hulsmeyer V.I., Fischer A., Mandigers P.J., De Risio L., Berendt M., Rusbridge C., Bhatti S.F., Pakozdy A., Patterson E.E., Platt S. (2015). International Veterinary Epilepsy Task Force’s current understanding of idiopathic epilepsy of genetic or suspected genetic origin in purebred dogs. BMC Vet. Res..

[10] Volk H.A., Matiasek L.A., Luján Feliu-Pascual A., Platt S.R., Chandler K.E. (2008). The Efficacy and Tolerability of Levetiracetam in Pharmacoresistant Epileptic Dogs. Vet. J..

[11] Potschka H., Fischer A., Löscher W., Volk H.A. (2023). Pathophysiology of drug-resistant canine epilepsy. Vet. J..

[12] Munana K.R., Nettifee-Osborne J.A., Papich M.G. (2015). Effect of Chronic Administration of Phenobarbital, or Bromide, on Pharmacokinetics of Levetiracetam in Dogs with Epilepsy. J. Vet. Intern. Med..

[13] Dewey C.W., Bailey K.S., Boothe D.M., Badgley B.L., Cruz-Espindola C. (2008). Pharmacokinetics of Single-Dose Intravenous Levetiracetam Administration in Normal Dogs. J. Vet. Emerg. Crit. Care.

[14] Patsalos P.N. (2004). Clinical Pharmacokinetics of Levetiracetam. Clin. Pharmacokin..

[15] Patterson E.E., Goel V., Cloyd J.C., O’Brien T.D., Fisher J.E., Dunn A.W., Leppik I.E. (2008). Intramuscular, Intravenous and Oral Levetiracetam in Dogs: Safety and Pharmacokinetics. J. Vet. Pharmacol. Ther..

[16] Lyseng-Williamson K.A. (2011). Spotlight on Levetiracetam in Epilepsy. CNS Drugs.

[17] Moore S.A., Muñana K.R., Papich M.G., Nettifee-Osborne J.A. (2011). The Pharmacokinetics of Levetiracetam in Healthy Dogs Concurrently Receiving Phenobarbital. J. Vet. Pharmacol. Ther..

[18] Segev G., Cortellini S., Foster J.D., Francey T., Langston C., Londoño L., Schweighauser A., Jepson R.E. (2024). International Renal Interest Society Best Practice Consensus Guidelines for the Diagnosis and Management of Acute Kidney Injury in Cats and Dogs. Vet. J..

[19] Cobrin A.R., Blois S.L., Kruth S.A., Abrams-Ogg A.C., Dewey C. (2013). Biomarkers in the assessment of acute and chronic kidney diseases in the dog and cat. J. Small Anim. Pract..

[20] Nabity M.B., Lees G.E., Boggess M.M., Yerramilli M., Obare E., Yerramilli M., Rakitin A., Aguiar J., Relford R. (2015). Symmetric Dimethylarginine Assay Validation, Stability, and Evaluation as a Marker for the Early Detection of Chronic Kidney Disease in Dogs. J. Vet. Intern. Med..

[21] Relford R., Robertson J., Clements C. (2016). Symmetric Dimethylarginine: Improving the Diagnosis and Staging of Chronic Kidney Disease in Small Animals. Vet. Clin. North Am. Small Anim. Pract..

[22] International Renal Interest Society (2023). IRIS Staging of Chronic Kidney Disease (Modified 2023). https://www.iris-kidney.com.

[23] Yau K., Burneo J.G., Jandoc R., McArthur E., Muanda F.T., Parikh C.R., Wald R., Weir M.A., Garg A.X. (2019). Population-Based Study of Risk of AKI with Levetiracetam. Clin. J. Am. Soc. Nephrol..

[24] Al-Quliti K., Alhujeily R.M. (2022). Acute Kidney Injury with Levetiracetam in Patient with Epilepsy: A Case Report. Case Rep. Clin. Med..

[25] Chau K., Yong J., Ismail K., Griffith N., Liu M., Makris A. (2012). Levetiracetam-Induced Severe Acute Granulomatous Interstitial Nephritis. Clin. Kidney J..

[26] Hurwitz K.A., Ingulli E.G., Krous H.F. (2009). Levetiracetam-Induced Interstitial Nephritis and Renal Failure. Pediatr. Neurol..

[27] De Risio L., Bhatti S., Muñana K., Penderis J., Stein V., Tipold A., Berendt M., Farqhuar R., Fischer A., Long S. (2015). International Veterinary Epilepsy Task Force Consensus Proposal: Diagnostic Approach to Epilepsy in Dogs. BMC Vet. Res..

[28] Muñana K.R., Thomas W.B., Inzana K.D., Nettifee-Osborne J.A., McLucas K.J., Olby N.J., Mariani C.J., Early P.J. (2012). Evaluation of levetiracetam as adjunctive treatment for refractory canine epilepsy: A randomized, placebo-controlled, crossover trial. J. Vet. Intern. Med..

[29] Dewey C.W. (2006). Anticonvulsant therapy in dogs and cats. Vet. Clin. North Am. Small Anim. Pract..

[30] Bilbrough G., Evert B., Hathaway K., Panagakos G., Robertson J., Yerramilli M. (2018). IDEXX Catalyst SDMA Test for In-House Measurement of SDMA Concentration in Serum from Dogs and Cats.

[31] Krimer P.M., Latimer K.S. (2011). Generating and Interpreting Test Results: Test Validity, Quality Control, Reference Values, and Basic Epidemiology. Duncan & Prasse’s Veterinary Laboratory Medicine: Clinical Pathology.

[32] Scobie C., Dean R., Stavisky J., Plüddemann A. (2026). Diagnostic Accuracy of Symmetric Dimethylarginine for Chronic Kidney Disease in Cats and Dogs: A Systematic Review. Vet. Rec..

[33] Gim S.Y., Song W.J., Youn H.Y. (2021). Use of Levetiracetam in Epileptic Dogs with Chronic Kidney Disease: A Retrospective Study. Vet. Sci..

[34] De Santis F., Boari A., Dondi F., Crisi P.E. (2022). Drug-Dosing Adjustment in Dogs and Cats with Chronic Kidney Disease. Animals.

[35] Packer R.M., Nye G., Porter S.E., Volk H.A. (2015). Assessment into the Usage of Levetiracetam in a Canine Epilepsy Clinic. BMC Vet. Res..

[36] Potschka H., Fischer A., Löscher W., Patterson N., Bhatti S., Berendt M., De Risio L., Farquhar R., Long S., Mandigers P. (2015). International Veterinary Epilepsy Task Force Consensus Proposal: Outcome of Therapeutic Interventions in Canine and Feline Epilepsy. BMC Vet. Res..

