# Supplementary material for: Short-Term Renal Effects of Add-On Levetiracetam in Client-Owned Dogs with Idiopathic Epilepsy Receiving Phenobarbital: A Four-Week Prospective Study
Source: Vet Sci. 2026 Aug 23;13(9):852. doi: 10.3390/vetsci13090852 (PMC13611570; doi:10.3390/vetsci13090852)
Supplement: Supplementary file 1 [file vetsci-13-00852-s001.zip › vetsci-4490218-supplementary.pdf]

Figure S1. Representative magnetic resonance (MR) images from one dog fulfilling the IVETF Tier II diagnostic confidence level. (a) Transverse T2-weighted image and (b) sagittal T2-weighted image showing no evidence of structural intracranial abnormalities.

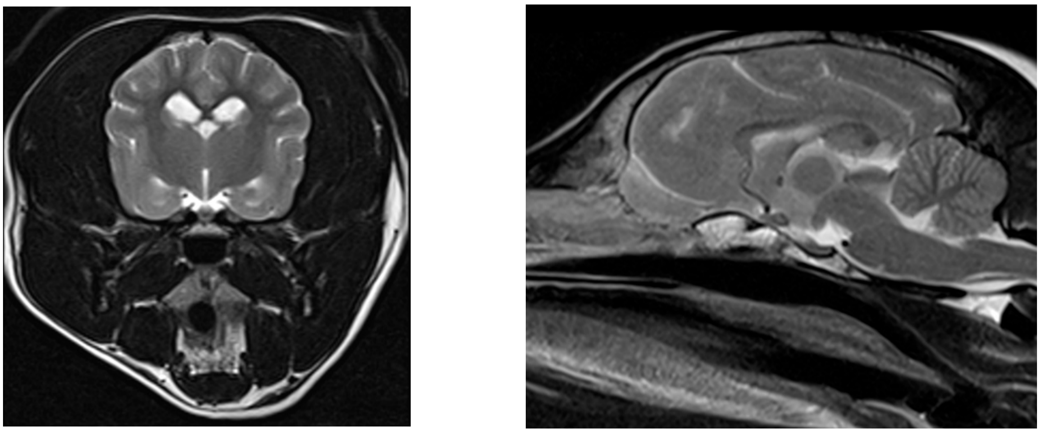

(a) (b)

Three dogs fulfilled the IVETF Tier II diagnostic confidence level based on advanced diagnostic evaluation. Representative MR images from one of these dogs are provided in Supplementary *Figure S1* and showed no evidence of structural intracranial abnormalities.

Table S1. Confidence intervals for renal biomarkers and serum biochemical parameters during the 4-week treatment period.

**Variable**	**Day 0**	**Day 14**	**Day 28**
SDMA, CI of median	10.00–14.00	10.00–13.00	10.00–14.00
BUN, 95% CI of mean	14.94–20.89	13.14–21.86	16.00–24.50
Serum creatinine, 95% CI of mean	0.887–1.172	0.889–1.256	0.958–1.227
Albumin, 95% CI of mean	3.129–3.721	3.111–3.472	3.070–3.563
Phosphorus, 95% CI of mean	3.469–4.114	3.546–3.987	3.564–4.136

CIs are presented for the median of SDMA and the means of BUN, serum creatinine, albumin, and phosphorus. For SDMA, the nonparametric confidence interval for the median generated by GraphPad Prism had an actual confidence level of 96.14% because of the discrete nature of the data and sample size. For the remaining variables, two-sided 95% confidence intervals of the means are presented. CI = confidence interval; SDMA = symmetric dimethylarginine; BUN = blood urea nitrogen.
